# Health Patterns across Adulthood: An Age-Based Investigation of the Nutritional Status, Homocysteine, and CoQ10 of Bank Staff

**DOI:** 10.3390/clinpract14020034

**Published:** 2024-03-14

**Authors:** Markus Schauer, Susanne Mair, Mohamad Motevalli, Derrick Tanous, Martin Burtscher, Katharina Wirnitzer

**Affiliations:** 1 Department of Sport Science, University of Innsbruck, 6020 Innsbruck, Austriamohamad.motevalli@uibk.ac.at (M.M.);; 2 Department of Research and Development in Teacher Education, University College of Teacher Education Tyrol, 6010 Innsbruck, Austria; 3 Research Center Medical Humanities, University of Innsbruck, 6020 Innsbruck, Austria; 4 Department of Pediatric Oncology and Hematology, Charité—Universitätsmedizin Berlin, 13353 Berlin, Germany

**Keywords:** occupation, homocysteine, coenzyme Q10, vitamin, mineral, nutrition, physical activity, lifestyle, metabolic health, public health

## Abstract

Background: This study aimed to evaluate age-specific variations in the blood levels of micronutrients, homocysteine, and CoQ10, along with physical activity (PA) patterns, among 123 Austrian adult bankers in operational and frontline roles (mean age: 43 years; 50% female). Methods: Blood analysis was conducted to assess micronutrients and the serum concentrations of homocysteine and CoQ10. The micronutrient values in whole blood were compared to sex-specific reference ranges and categorized as below, within, or above them. The Global Physical Activity Questionnaire was utilized to assess PA patterns. Participants were classified as young adults (18–34 years), middle-aged adults (35–49 years), and older adults (50–64 years). Results: Significant age-based differences were found in participants’ mean homocysteine levels (*p* = 0.039) and homocysteine categories (*p* = 0.034), indicating an increasing prevalence of hyperhomocysteinemia with age. No significant difference between age categories was observed for sex, BMI, diet types, PA levels, sedentary behavior, and CoQ10 (*p* > 0.05). There was no significant age-based difference in the blood concentrations of most minerals and vitamins (*p* > 0.05), except for magnesium among females (*p* = 0.008) and copper among males (*p* = 0.042). Conclusion: The findings offer initial evidence of the age-related differences in the health status of adult bankers, providing insights for customized approaches to occupational health that support the importance of metabolic health and overall well-being across adulthood.

## 1. Introduction

As a worldwide health concern, non-communicable diseases (NCDs; including cardiovascular diseases, cancer, metabolic disorders, musculoskeletal dysfunctions, and mental health concerns) play a pivotal role in global morbidity and mortality [1]. Evidence indicates that sedentary lifestyles are significantly linked to the rise in prevalence of NCDs [2]. In addition, dietary-associated health conditions (including various NCDs) are known to be key factors of morbidity and mortality [3,4]. For instance, while cardiovascular disease is recognized as the leading cause of death among people aged above 70, cancer is the primary cause of mortality among adult populations [3]. While adopting a healthy lifestyle can serve as a preventive and even therapeutic approach to managing NCDs among different populations [5], the proactive monitoring of a person’s health and nutritional status is crucial for the timely identification of early signs of potential health risks, navigating health-related trajectories, and designing targeted health-promoting interventions.

Biological age is considered a risk factor for a variety of chronic health conditions, including those related to metabolic health [6]. The aging process is associated with a decline in metabolic rate, alterations in body composition, and changes in glucose and lipid metabolism, which can independently increase a person’s risk of metabolic disorders, cardiovascular diseases, and obesity [6]. In addition, the age-related decline in mitochondrial function and cellular metabolism can further exacerbate the abovementioned risks [7]. Over the past few decades, there has been increasing attention towards investigating age-related variations in nutritional and health status, with the aim of developing tailored health approaches. Throughout different stages of adulthood, data show that individuals experience distinct dynamics in their health behaviors and status that profoundly impact their overall well-being. Young adults, for example, usually exhibit elevated levels of energy metabolism while encountering fewer health limitations [8,9], and their health is potentially influenced by social factors, peer stimulus, and the pursuit of individual objectives [10]. However, the health condition of middle-aged adults may be associated with stress management, weight maintenance, and the initial signs of chronic diseases, possibly due to the demands of their busy work and family routines [11,12]. Meanwhile, older adulthood is a period marked by a multitude of age-related physiological changes, including a decrease in muscle mass and bone density, alongside metabolic abnormalities, which significantly impact health and well-being [13,14]. These facts highlight that comprehending the potential variations in health and nutritional status attributed to age-related differences is essential for formulating health-promoting interventions.

Occupational health is a field of study and practice that focuses on ensuring the physical, mental, and social well-being of workers in their workplace environment [15,16]. Evidence indicates that the sedentary nature of most occupations is associated with an increased likelihood of NCDs and the prevalence of obesity [16]. According to the Austrian Health Survey 2019, approximately half of the Austrian working-age population were mostly physically inactive during worktime [17]. As a group susceptible to health risks, bank employees often spend extended hours seated at desks or in front of computer screens, tackling mentally demanding responsibilities that could potentially cause them to develop unhealthy eating patterns and lead to adverse health consequences [18]. Therefore, the substantial influence of the work environment on health and well-being emphasizes the requirement for proactive health monitoring within various working demographics.

Nutritional assessment is a clinical approach that is conducted for the diagnosis of chronic conditions and the comprehensive evaluation of health status [19,20,21]. The significance of micronutrients, particularly vitamins and minerals, is highlighted in their key roles across various physiological mechanisms, spanning DNA generation, energy metabolism, antioxidant defense, immune activity, hormonal regulation, and neurotransmitter synthesis, contributing to biological homeostasis and a person’s overall health status [20,22]. Clinical evidence shows that deficiencies in micronutrients can cause a wide range of health problems including metabolic disorders, ranging from deficiencies like anemia to more complex conditions such as diabetes and cardiovascular disease [20]. For example, when considering obesity as a primary risk factor for NCDs [23], evidence reports that inadequate levels of blood vitamin D are associated with an increased risk of obesity and metabolic syndrome [24,25]. In addition, hypertension and cardiovascular disorders are reported to be associated with deficiencies in some minerals, such as magnesium, potassium, and calcium [26]. Considering these findings, it is essential to underline the importance of monitoring micronutrient status and addressing their deficiencies to promote overall health and well-being.

Clinical data indicate that biomarkers, as health indicators, are linked to the risk factors of chronic health conditions. Homocysteine, a byproduct of methionine biosynthesis, is known as a significant indicator of cardiovascular diseases, with the potential to impact the development of atherosclerosis [27,28]. Research has revealed that micronutrients such as vitamins B_6_, B_9_, and B_12_ play a critical role in homocysteine metabolism, and deficiencies in these vitamins are linked to disturbances in the metabolic process of homocysteine, leading to elevated homocysteine levels and their associated adverse health effects [27,28]. Coenzyme Q10 (CoQ10) is an antioxidant and a critical element of cellular respiration, playing a role in neutralizing free radicals and thereby protecting cells against oxidative damage [29,30]. Similar to homocysteine, CoQ10’s biogenesis is linked to several micronutrients, including B-group vitamins and specific minerals such as zinc, iron, and copper [30,31]. These facts indicate the significance of micronutrients in the metabolic biosynthesis of health biomarkers, particularly homocysteine and CoQ10, which further highlights the interplay between nutritional status and overall health and well-being.

Despite the growing awareness of the significance of adopting a healthy lifestyle and regularly monitoring one’s nutritional status, there remains a research gap in investigating differences in micronutrients and health status across various stages of adulthood within the context of occupational health. The present study aims to address this gap by focusing on the age-related differences in blood micronutrient levels, PA levels, sedentary behaviors, and CoQ10 and homocysteine levels among bank employees, a population known for its vulnerability to health issues. The overarching objective is to offer valuable perspectives that can be utilized in designing personalized nutritional approaches aimed at promoting health benefits for individuals working in sedentary settings.

## 2. Materials and Methods

### 2.1. Study Design and Participants

A cross-sectional design was used in the present investigation. Individuals working in Austrian banks located in the western federal state of Tyrol, within the age range of 18 to 64 years, were invited to participate in the study on a voluntary basis. Any adult individual working for a bank in Tyrol was eligible to take part in this study. Ethical endorsement for the research procedure was granted by the independent Ethics Board of the Medical University of Innsbruck (Ethikkommission Nr: 1136/2022; approval date: 9 January 2023), in alignment with worldwide scientific and ethical principles, encompassing the Good Clinical Practice guidelines and the Helsinki Declaration. Data collection occurred within the standard, optional corporate health program for employees of Tyrolean banks, carried out at the Biogena Diagnostics Point Tyrol throughout December 2019 and January 2020. Individuals were briefed about the study’s aims, methods, possible advantages, and drawbacks, and their formal consent was obtained prior to their involvement in the research. The invitations emphasized the voluntary nature of participation, making it clear that joining the study was entirely optional. Participants were directly informed about their prerogative to exit the study at any time without having to give a reason. No compensation was offered for their participation in this research. Initially, a total of 280 individuals, from 73 different bank offices/units across 12 cities in Austria, were registered for participation. This sample was out of an estimated population of around 4500 bankers in the state of Tyrol, Austria. Before conducting statistical analysis, we excluded one participant who had been diagnosed with stomach cancer, a condition that may significantly affect their nutritional status. The final analysis included 123 bank employees who fulfilled all necessary assessments (including blood tests) and formed a complete and valid data set. The study maintained the confidentiality of individuals’ data at all times, implementing steps to anonymize and securely manage all gathered data. Meticulous ethical considerations were embraced to uphold the rights, privacy, and welfare of the participants involved in this study. An artificial intelligence (AI) tool, Grammarly (Grammarly Inc., San Francisco, CA, USA), was used to promote scientific language and proofread the final report.

### 2.2. Measurements and Classifications

In this study, two main types of measurement were conducted. Firstly, blood samples were collected at the Biogena Diagnostic Point Innsbruck for evaluating participants’ micronutrients (including minerals and vitamins), homocysteine, and CoQ10 levels. The analysis of the blood samples was conducted by GanzImmun (Mainz, Germany), adhering to standardized laboratory evaluation procedures [32]. Secondly, a questionnaire consisting of multiple modules was distributed 1. to collect general information, including sociodemographic characteristics, anthropometric measurements, employment-related particulars, disease history, and diet-type preferences and 2. to evaluate physical activity (PA) levels and sedentary behaviors. The study questionnaire is available as supplementary material. Aligned with the current trends in automated banking in Austria, the majority of participants were in operational or frontline positions, with a similar work style in a tech-driven environment, often spending long hours in front of screens. A printed version of the questionnaire was completed by participants in the laboratory within 20 min.

**Figure 1:**
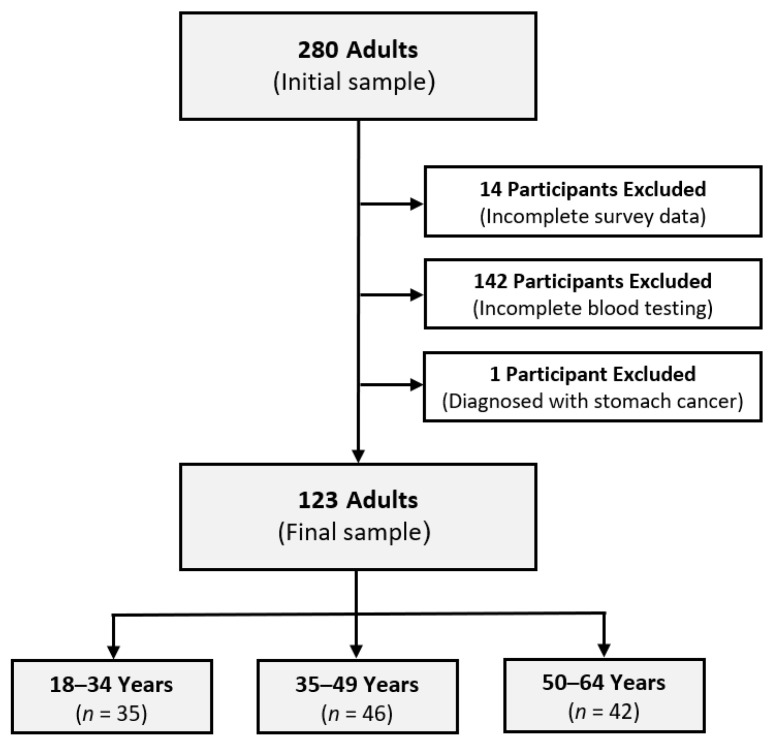
Flowchart of the study and classification of the participants.

To examine age-related differences in the study variables, participants were classified into three discrete age cohorts: 18–34 years old (*n* = 35), 35–49 years old (*n* = 46), and 50–64 years old (*n* = 42). In addition, self-reported body weight and height values were used to compute their body mass index (BMI; kg/m^2^). In accordance with the adult BMI classifications outlined by the World Health Organization [33], participants were sorted into four subgroups based on their BMI: underweight (<18.5 kg/m^2^), normal weight (18.5–24.9 kg/m^2^), overweight (25–29.9 kg/m^2^), and obese (≥30 kg/m^2^). Participants were also categorized into two groups based on the diet types they reported in the questionnaire: 1. omnivores (those without specific dietary limitations) and 2. vegetarians (those who exclude meat- and fish-based items from their diet) or vegans (those who exclude all foods and ingredients sourced from animals from their diet) [34,35]. Figure 1 shows the study flowchart.

### 2.3. Blood Sampling and Laboratory Methods

The process of blood collection adhered to standardized laboratory techniques to ensure the preservation of the samples’ integrity. After a fasting period of 12 h, venous blood samples were obtained between 8:00 and 11:00 using anticoagulant tubes. Each blood specimen was divided into two parts, with one portion allocated for serum extraction. After centrifugation (3 min, at room temperature, with 3000 revolutions per minute), the serum was segregated from the red blood cells, placed into cryovials, and dispatched to the GanzImmun laboratory for further analysis within two hours.

The assessment of micronutrient levels (including potassium, calcium, magnesium, copper, iron, zinc, selenium, manganese, molybdenum, vitamin B_6_, vitamin B_9_ (folate), vitamin B_12_, and vitamin D) was accomplished utilizing whole blood samples, adhering to standardized laboratory procedures [32]. Since numerous micronutrients primarily reside within blood cells, a comprehensive analysis of whole blood’s content provides more meaningful insights than measuring serum samples [32,36,37]. Since a significant part of the biochemical reactions involving minerals and trace elements occurs primarily within cells [38], serum values may not necessarily reflect micronutrient concentrations at the cellular level. Thus, it appears that by analyzing blood cells, which primarily originate from vigorously metabolizing bone marrow, a more precise approach is adopted to measure the metabolic conditions of these micronutrients [32]. Consequently, a thorough evaluation of blood cells was executed to achieve a representation of participants’ micronutrient levels.

The quantification of CoQ10 levels was achieved using high-performance liquid chromatography (HPLC) coupled with ultraviolet detection. The CoQ10 values acquired from the blood samples were adjusted relative to participants’ cholesterol levels. There is a well-established association between CoQ10 and cholesterol, suggesting the involvement of CoQ10 in the electron transport chain and its role in cholesterol synthesis [39]. In addition, given the variation in cholesterol metabolism among individuals [39], it is important to adjust CoQ10 levels in response to changes in cholesterol, which contributes to improving the accuracy of measurements. Therefore, the CoQ10 values were divided by their corresponding cholesterol levels to compute a CoQ10/cholesterol ratio.

Chemiluminescence immunoassay (CLIA) kits (Beijing O&D Biotech Co., Ltd., Beijing, China), with a detection range of 1 to 50 µmol/L, were used to evaluate the serum homocysteine concentration. The analysis, undertaken by a senior laboratory technician, was conducted using the Centaur XPT analyzer (Siemens Healthineers, Forchheim, Germany) and involved reagents. The homocysteine samples demonstrated an intra-assay coefficient of variation of 3.9% and an inter-assay coefficient of variation of 5.8%. Participants were grouped into three categories based on their homocysteine levels: under 10 µmol/L, between 10 and 15 µmol/L, and above 15 µmol/L [40].

### 2.4. Physical Activity

The participants’ PA levels and sedentary time were assessed using the Global Physical Activity Questionnaire (GPAQ). As a validated and widely used tool developed by the World Health Organization (WHO), the GPAQ collects comprehensive data on PA behavior [41,42]. Detailed information on the validation of the GPAQ for German-speaking adult populations can be found in another study [42]. Participants were asked to fill out the GPAQ survey, providing details about their PA during a typical week. The questionnaire covers work, transport, recreation, and sitting activities. The intensity of each activity type is quantified using metabolic equivalents of tasks (METs), which represent the ratio of the metabolic rate during a specific activity to the resting metabolic rate, with one MET being equivalent to the energy expenditure at rest. To analyze PA levels, participants were required to report the duration and frequency of activities classified as vigorous-intensity, moderate-intensity, and walking. By applying this specific computation approach, PA levels were calculated and classified as low PA, moderate PA, or high PA, in line with the WHO’s weekly PA recommendations [41,43]. Additionally, the survey allowed participants to document their sedentary periods, quantified in hours every week, which included the periods of sitting or resting throughout a usual day, except for sleep hours.

### 2.5. Statistical Analysis

The statistical software R version 4.1.1 (R Foundation for Statistical Computing, Vienna, Austria) was used to conduct all statistical analyses. A power analysis was conducted to determine the sample size, resulting in a finding that 120 participants were needed based on the experimental design (two sex groups and three age groups), with an assumed medium effect size of 0.28, a power of 0.80, and an alpha level of 0.05. Exploratory analysis was performed using descriptive statistics, including mean values and standard deviation (SD) or median, range, and interquartile range (IQR). The normality of numerical variables was evaluated using the Shapiro–Wilk test. For nominal scale data, Pearson chi-square tests (χ^2^) were used to examine the association of age categories with sex, BMI levels, PA levels, homocysteine levels, and diet types. Kruskal–Wallis tests were applied for ordinal and metric-scaled variables, using t or F distributions, ordinary least squares, and standard errors (SE), as well as R^2^ to assess the association of age categories with height, weight, BMI, homocysteine, CoQ10, sedentary time, and adjusted CoQ10. Visual depictions in the form of box plots, accompanied by 95% confidence intervals, were designed to show the potential disparities in the blood variables associated with age groups. Likert plots were created to show the variation of micronutrients across age groups based on their sex-specific reference ranges [32]. The predetermined level for statistical significance was set as *p* ≤ 0.05.

## 3. Results

**Figure 2:**
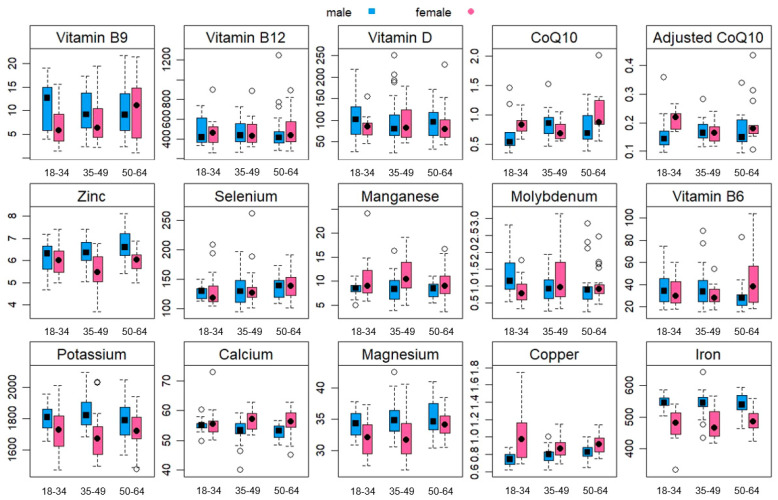
Box plots demonstrating the differences between age categories (18–34, 35–49, and 50–64 years) across blood variables. This presentation of results is based on median and quantile values, with statistical outliers shown as circles.

A sample of 123 individuals (62 females and 61 males) with a median age of 43 years underwent our final statistical analysis. The prevalence of underweight participants was 6%, while 33% of participants were classified as overweight or obese. A majority of the participants (93%) declared their adherence to an omnivorous diet, whereas 7% identified as vegetarians or vegans. Concerning PA levels, it was observed that 61% of the participants had a high PA level, while moderate and low PA levels were evident in 21% and 18% of the cohort, respectively. Significant differences were found in both the mean homocysteine levels and homocysteine categories among subgroups (*p* < 0.05), indicating a rise in mean homocysteine values as age advances (*p* = 0.039) and a higher prevalence of homocysteine values exceeding 15 µmol/L within the older group (14%) compared to the younger group (3%) (*p* = 0.034). No significant difference between age categories was found for sex, body weight, height, BMI, BMI categories, diet type, PA levels, sedentary time, absolute CoQ10 values, or adjusted CoQ10 (*p* > 0.05). Table 1 shows a summary of participants’ data in terms of the study variables across the three age categories. Figure 2 shows the differences between age categories for different blood variables.

Among female participants, a significant difference was observed between age categories in their blood levels of magnesium, indicating a gradual increase in magnesium concentration (from 31.9 ± 3.1 to 34.2 ± 1.9 mg/L) as age advances (*p* = 0.008). In the female cohort, no significant difference between age categories was found in the blood concentrations of other micronutrients (*p* > 0.05). Among male participants, a significant difference between age categories was detected in their blood levels of copper (*p* = 0.042), while no association was found between age and the blood concentrations of other micronutrients (*p* > 0.05). Table 2 and Table 3 represent the blood micronutrient levels of females and males across age categories.

**Figure 3:**
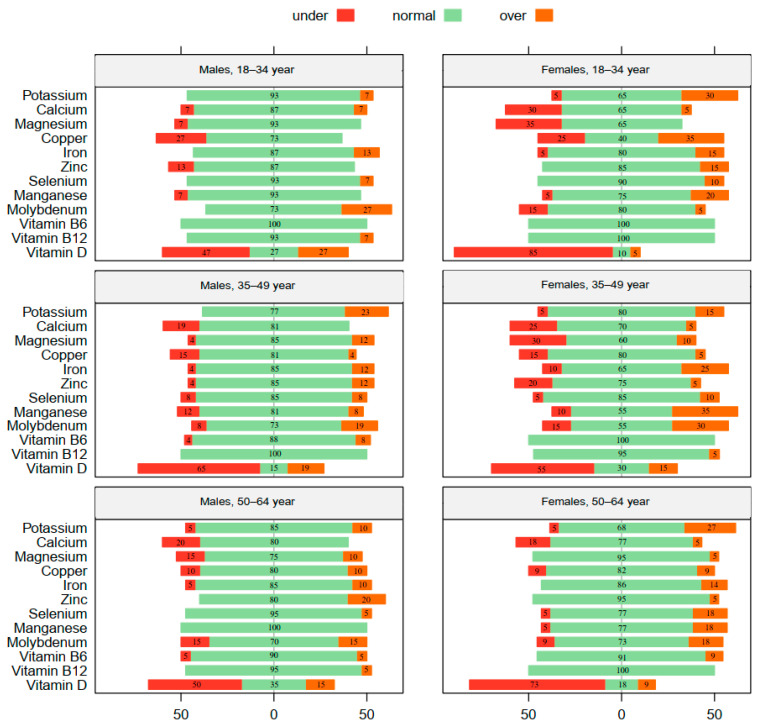
Likert plots displaying the extent of the variation in micronutrient mean values from the sex-specific reference norms for German-speaking populations [32]. The variations are presented as percentages from the zero point and are color-coded into three categories: red (under the reference range), green (within the reference range), and orange (over the reference range). Data are classified based on three age groups: 18–34, 35–49, and 50–64 years old, for males and females separately.

Figure 3 displays the extent of the variation of the participants’ micronutrient values from sex-specific reference norms, differentiated by age categories. The blood levels of vitamin D reveal that most females, particularly those in the younger (18–34 years) and older (50–64 years) age groups, exhibited vitamin D levels below the standard range. Among males, however, a more pronounced deficiency in vitamin D was observed among participants in the middle age category (35–49 years). Irrespective of their vitamin D levels, most males and females displayed a normal blood micronutrient level, predominantly falling within the standard reference range.

## 4. Discussion

This study aimed to conduct an age-based comparison of the micronutrient status, homocysteine and CoQ10 blood concentrations, PA levels, and sedentary behavior, across three age-based categories of adulthood, of Tyrolean bank employees. The most notable findings are as follows: (i) while 6% of participants were classified as underweight, the prevalence of overweight/obesity was 33%, with no significant differences observed between age categories; (ii) although statistically insignificant, the prevalence of a high PA level was greater among younger adults (aged 18–35 years), while the prevalence of a low PA level was greater among older participants (aged 50–65 years); (iii) there was a significant difference in both the mean homocysteine levels and homocysteine categories among the study groups, including an increase in the prevalence of hyperhomocysteinemia (homocysteine > 15 µmol/L) as age advances; (iv) no significant difference between age categories was found for sex, body weight, height, BMI, BMI categories, diet types, PA levels, sedentary time, or CoQ10 blood levels; (v) among females, while a significant difference was observed between age categories in their blood levels of magnesium, no significant age-based difference was found in their blood concentrations of other micronutrients (including calcium, potassium, iron, zinc, copper, selenium, molybdenum, manganese, vitamin B_6_, vitamin B_9_, vitamin B_12_, and vitamin D); (vi) among males, except for copper, no significant association was found between age and their blood concentrations of micronutrients; and (vii) the majority of females and males displayed a normal blood micronutrient status, predominantly falling within the standard reference range; however, most participants exhibited a deficiency in vitamin D.

### 4.1. Micronutrients

As documented in both the DGE Nutrition Report [44] and the Austrian Nutrition Reports of 2012 and 2017 [3,4], adults often face challenges in achieving an adequate intake of a variety of micronutrients (including vitamin A, vitamin D, vitamin B_9_, vitamin B_12_, magnesium, potassium, calcium, and iodine) through their typical diet. This challenge arises from several factors, including interindividual variations in micronutrient homeostasis, dietary habits, food availability, and adults’ levels of nutritional knowledge awareness [3,4,44]. The present research also examined participants’ micronutrient levels to gain a better understanding of age-related trends in the blood concentrations of minerals and vitamins. Accordingly, no significant age-based differences were found in the blood concentrations of most micronutrients when analyzed separately for males and females. The absence of significant age-based differences in the blood concentrations of most micronutrients in this study could be attributed to a complex interplay of confounding factors, such as physiological and dietary variables. Additionally, methodological factors (including the laboratory techniques used for assessing micronutrients), and subject-related factors (such as socioeconomic status, race, and ethnicity) should also be considered as potential interactive variables. Therefore, further research is needed to explore these dynamics comprehensively. Compared to reference norms [32], most females and males displayed normal blood micronutrient levels, predominantly falling within the standard reference range.

Most participants in all age categories exhibited a deficiency in vitamin D, which is consistent with findings from a similar Austrian study on adult populations [45], as well as numerous international investigations [46,47,48]. The present study showed that most females, especially those in the younger (18–34 years) and older (50–64 years) age categories, demonstrated vitamin D levels below the reference range. Among males, however, a more evident deficiency in vitamin D was observed in middle-aged adults (35–49 years). Evidence indicates that factors such as reduced sunlight exposure may contribute to a lower cutaneous synthesis of vitamin D [49]. It is important to consider that the data collection for the present study was conducted during the winter months (December and January), when there is less sunlight compared to the summer. However, existing research indicates that the average serum concentration of vitamin D during winter in Austrian populations tends to be higher than that in most European countries [50]. While data indicate the universal nature of vitamin D deficiency and emphasize the need for tailored strategies to tackle this issue [51], the validity of the cutoff values utilized to classify vitamin D deficiencies among diverse population groups is also a matter of consideration [45,52]. Altogether, these results emphasize the significance of assessing nutrient levels and highlight the necessity for customized strategies to tackle micronutrient inadequacies, with a particular focus on vitamin D, which emerges as the primary concern across all age groups during adulthood. These data also highlight the importance of age differences when addressing micronutrient deficiencies in public health strategies. Tailoring public health interventions to address these age-specific patterns is crucial, and age-specific approaches can enhance the effectiveness of public health initiatives, ensuring that resources are directed to where they are most needed.

### 4.2. Homocysteine and CoQ10

In the present study, the assessment of serum homocysteine and CoQ10 levels was conducted to monitor health status and highlight the significance of age-related considerations within the framework of occupational health. Hyperhomocysteinemia is defined as increased homocysteine levels above 15 μmol/L, which is associated with several diseases, and predominantly an increased risk of cardiovascular disorders [40,53,54]. In the total sample, 9% of participants exhibited hyperhomocysteinemia, while 39% of participants fell within the borderline risk range (with blood homocysteine levels between 10 and 15 μmol/L). Consistent with this finding, data from a comparable investigation indicate that approximately 7% of adults in the German population exhibited hyperhomocysteinemia [55]. In the present study, a significant difference in the prevalence of hyperhomocysteinemia (homocysteine > 15 µmol/L) was observed among different age categories, as older participants exhibited a higher prevalence (14%) compared to the young (3%) and middle-aged (9%) groups. Consistently, data obtained from large-scale studies provide compelling evidence that age serves as a significant indicator of homocysteine concentrations and the prevalence of hyperhomocysteinemia [56,57,58,59], which has provoked recommendations that the reference ranges for serum homocysteine concentrations should be adjusted to account for age-related variations [60]. However, it should be considered that the blood levels of homocysteine are strongly influenced by genetic factors, affected by both rare genetic variants and commonly occurring genetic polymorphisms [61]. Therefore, while age is a key factor in the prevalence of hyperhomocysteinemia, a further comprehensive understanding of the interplay between age and genetic determinants is essential for a more accurate interpretation of homocysteine levels and their associated metabolic consequences.

In the present study, no significant difference was found between age categories in both the absolute and adjusted levels of CoQ10. While there has not been a specific study investigating the homocysteine and CoQ10 levels among bank employees, an investigation conducted on Brazilian bank employees [62] did identify age as a significant indicator of metabolic syndrome, a well-documented health parameter, revealing a gradual increase in the prevalence of metabolic syndrome with advancing age across four categories of adulthood [62]. In this regard, various factors (including individual variations in hormonal status, metabolic processes, and lifestyle behavior) have been reported as key mediators of metabolic syndrome [63]. Results from an Indian study indicate a noteworthy trend in the prevalence of chronic diseases among bank employees, showing a significant increase with advancing age across adulthood [64]. Accordingly, the prevalence of chronic diseases was 25% among young bank employees (aged 20–30 years) and this increased by 15–20% in each successive 10-year age category, reaching 90% among those aged 50–60 years [64]. However, it should be considered that the biological state of professional cohorts can be shaped by both individuals’ inherent biological tendencies and the prolonged effects of their work environment [65]. In general, it is worth emphasizing that age-specific considerations in occupational health assessments necessitate further investigations that explore the interactions between additional health parameters while rigorously controlling for potential confounding factors. This includes a focus on how metabolic processes, lifestyle behaviors, and hormonal status interact with advancing age, particularly in the context of occupational health.

### 4.3. PA Levels and Sedentary Time

There is a growing body of evidence emphasizing the significant impact of sedentary behavior on morbidity and mortality risk [66,67,68]. The present study revealed that only 18% of bank employees reported a low PA, which is remarkably lower than the estimated prevalence of low PA levels among either European or Austrian adults [69]. In this regard, results from two different large-scale German studies show that only 20% of German adults reach the minimum PA recommendations [70] and that age is a significant indicator of daily engagement in moderate-to-vigorous PA in adulthood [71]. Results from the Austrian Health Interview Survey 2019 also indicate that the estimated prevalence of sufficient PA levels gradually decreases with advancing age [72]. Particularly, the prevalence of adequate PA levels stands at 31.9% among young adults, while it is 22.8% for middle-aged adults and 20.0% for older-aged adults [72]. In the context of occupational health, a study reported that approximately half of Brazilian bank employees were physically active [73]. However, it should be considered that differences in how PA is measured, the potential bias in self-reported data, and the possible impact of sociocultural, socioeconomic, and environmental factors may contribute to the reported levels of PA participation. Additionally, it is crucial to consider that certain profession-related factors could potentially influence PA data, including access to recreational spaces in the workplace or residential areas, the socioeconomic status of bank employees, and corporate involvement in wellness initiatives. The present results show an age-based difference (although insignificant) in PA levels, with the prevalence of a low PA level being notably higher among bank employees aged 50–64 years (29%) compared to young (11%) and middle-aged (13%) participants. While this finding can be attributed to the increasing likelihood of sedentary behavior and reduced PA with advancing age, it is important to note that potential age-related health considerations can also influence PA habits [74].

The banking profession typically involves extended periods of sitting and engaging in cognitively demanding tasks. Data show that prolonged sitting hours are linked to an elevated mortality risk [68]. The present study found that the amount of time participants spent performing sedentary behavior (42.5 h per week or 364 min per day) was lower when compared to the results of a comparable German study, where adults spent an average of 552 min per day sedentary [71]. Although the present study found no significant age-based difference in sedentary time, it is worth noting that middle-aged participants showed a tendency toward having less sedentary time (38.8 h per week) compared to both their younger (43.9 h per week) and older (45.3 h per week) colleagues. According to the results from two different investigations, the prevalence of physical inactivity among bank employees was documented as 60% [75] and 82% [76], without any significant difference between younger and older adults [76]. However, one study has reported that younger adults (aged 30–49 years) exhibited higher levels of physical inactivity compared to their older counterparts (those aged 50–69 years) [77]. In a study focusing on the obstacles to embracing healthy habits, a significant factor contributing to the increasing rate of physical inactivity was the lack of time available for exercise [78]. Results from a meta-analysis show that prolonged periods of sedentary behavior, as measured by sitting time, were associated with an increased risk of all-cause mortality, and, interestingly, regular PA appeared to mitigate this effect [79]. Therefore, developing age-specific health promotion initiatives while considering the distinct patterns of PA and sedentary behavior among different age groups seems crucial in designing tailored health-promotion programs to enhance the population’s overall well-being, particularly in the context of public health interventions. By addressing age-specific needs, public health efforts can effectively combat the risks associated with sedentary behavior, ultimately aiming to reduce morbidity and mortality rates among people engaged in sedentary occupations.

### 4.4. Anthropometry

Examining the prevalence of overweight/obesity among bank employees, the present study delved into potential contributing factors while comparing its findings with national and international data. It was found that the prevalence of overweight/obesity among bank employees was 33%, which is lower than the general prevalence of overweight and obesity among Austrian adults (51.1%) [80]. Possible factors contributing to the above difference could include variations in occupational activity levels, access to workplace wellness programs, individual lifestyle choices, as well as the limited sample size of the present study for a prevalence assessment. According to the results of studies from Brazil and Ghana, the prevalence of overweight and obesity among bank employees was documented as being higher than 50% [81,82,83]. This difference with the present finding may be explained by a complex interplay of genetic, socio-cultural, and behavioral factors that influence body composition and adiposity differently across various populations [84,85,86]. In addition, socioeconomic status (influenced by factors like financial income, access to healthcare, and education level) can significantly impact the prevalence of overweight/obesity by influencing individuals’ ability to maintain a healthier BMI [87]. The present results also revealed a marginal (but insignificant: *p* = 0.074) difference between age categories, where the prevalence of overweight/obesity among the older group (48%) was higher than that among the young (14%) and middle-aged (35%) participants. Consistent with the present findings, data from a 2019 national report showed that there is a gradual increase in the prevalence of overweight and obesity across four age categories in adulthood, starting from 29.4% (among people aged 15–30 years) to 65.8% (among those aged 60–75 years) [80]. Comparable findings were also documented in Austrian investigations carried out among school teachers [88], university students [89], and university professors [90]. The age-associated difference in the prevalence of excess body weight could potentially be attributed to a combination of various factors, including a decreased metabolic rate, evolving lifestyles, and dietary habits [91]. However, it should be considered that relying on self-reported weight and height measurements may compromise the precision of BMI values and, subsequently, impact the accuracy of BMI classifications. These data provide valuable insights, with significant implications for public health. Strategies focusing on public health promotion should prioritize preventive measures, including promoting healthier lifestyle behaviors. Additionally, initiatives such as nutritional education and stress management programs can play a crucial role in improving the metabolic health and overall well-being of a target population.

### 4.5. Limitations and Strengths

This study had some limitations, including its cross-sectional design, which limits the establishment of causality between study variables and health outcomes. In addition, reliance on a survey-based methodology, particularly self-reported PA data, despite the questionnaire being validated, might be linked to the possibility of recall and reporting biases, which could result in the underreporting or overreporting of data. In addition, despite our efforts to collect data—especially by providing clear measuring and reporting instructions—relying on self-reported weight and height measurements may compromise BMI precision and, consequently, affect our classification accuracy. This study’s exclusive focus on bank employees may also limit the generalizability of its findings to other occupational sectors. Moreover, influential factors like socioeconomic status and specific job roles, which could act as confounding variables, were not factored into this analysis. Despite these limitations, the study’s strength lies in its thorough examination of multifaceted health parameters, which enhances our comprehension of the various factors of the health profile of Austrian bank employees. Furthermore, the study’s emphasis on age-specific analyses recognizes the potential for tailored considerations in assessing health outcomes. This study’s findings offer new insights into age-related differences in the health status of adult populations working in sedentary occupations, supporting the importance of tailored approaches to promote metabolic health and overall well-being across adulthood.

### 4.6. Implications

The present findings may contribute to our understanding of the potential implications of public health initiatives and workplace programs, particularly in the field of occupational health. Health promotion efforts should consider the lifestyle requirements of bank employees based on their age. While health approaches targeting young adults should predominantly emphasize the long-term benefits of establishing healthy habits in early life, health promotion strategies for middle-aged adults should incorporate time-efficient exercise routines and stress reduction techniques. Health behaviors among older adults may, therefore, focus on maintaining cognitive health, managing chronic conditions, and social engagement, with a particular emphasis on safety considerations, flexibility exercises, and mental stimulation. Focusing on this, regular health screenings and educational programs could empower bank employees through individualized health information and insights. These programs have the potential to increase knowledge regarding the nutrient deficiencies and lifestyle-related factors that contribute to cardiovascular risk, ultimately encouraging a culture of proactive health maintenance. Future studies could utilize longitudinal research methodologies to uncover the chronological associations between job-related factors, health indicators, and well-being consequences, offering more profound insights into how workplace variables influence health outcomes over time. Moreover, expanding the scope of investigation to encompass other occupational sectors would enable more extensive comparisons and a holistic comprehension of the variations in health parameters across diverse work settings. Assessing the efficiency of workplace interventions, whether focused on enhancing PA, dietary practices, or cardiovascular health, would also aid in developing evidence-backed approaches suitable for broader implementation.

## 5. Conclusions

Compared to the existing body of literature focusing on the Austrian and German populations, the bank employees in the present study exhibited optimal levels of PA (assessed using the GPAQ developed by the WHO) and favorable blood micronutrient profiles (determined through a comprehensive whole blood analysis). Significant differences in both mean homocysteine levels and homocysteine categories were observed among the age groups. No significant difference was observed between young, middle-aged, and older bank employees in terms of their PA habits, sociodemographic factors, CoQ10 levels, and most blood micronutrient concentrations. These findings provide initial evidence of the potential age-related variations in health and micronutrient status among a particular occupational group. These insights shed light on the potential implications for the field of occupational health, particularly in the development of customized strategies to beneficially impact health and overall well-being.

## Figures and Tables

**Table 1:** Summary of participants’ data based on three age categories.

		Total (*n* = 123)	18–34 Year (*n* = 35)	35–49 Year (*n* = 46)	50–64 Year (*n* = 42)	Statistics and *p*-Values
Sex	male female	50% 50%	43% 57%	57% 43%	48% 52%	χ^2^_(2)_ = 1.58, *p* = 0.453
Body Weight (kg)		71 (43–114)	65 (43–114)	74 (50–99)	72 (45–114)	F_(2, 120)_ = 2.10, *p* = 0.126
Height (cm)		173 (154–190)	170 (157–190)	175 (156–190)	172 (154–186)	F_(2, 120)_ = 1.51, *p* = 0.224
BMI (kg/m^2^)		23.6 (16.9–40.4)	22.7 (16.9–40.4)	23.8 (17.3–32.3)	24.2 (17.6–36.0)	F_(2, 120)_ = 3.00, *p* = 0.053
BMI Levels	<18.5	6%	9%	4%	5%	χ^2^_(6)_ = 11.51, *p* = 0.074
	18.5–24.9	61%	77%	61%	48%	
	25.0–29.9	28%	11%	33%	38%	
	≥30.0	5%	3%	2%	10%	
Homocysteine (µmol/L)		10.57 ± 4.30	9.73 ± 2.59	10.11 ± 4.10	11.77 ± 5.36	F_(2, 120)_ = 3.35, *p* = 0.039
Homocysteine Levels	<10	52%	60%	63%	33%	χ^2^_(4)_ = 10.43, *p* = 0.034
	10–15	39%	37%	28%	52%	
	>15	9%	3%	9%	14%	
CoQ10 (mg/L)		0.82 ± 0.28 *	0.73 ± 0.28	0.81 ± 0.22	0.89 ± 0.34	F_(2, 79)_ = 1.92, *p* = 0.153
Adjusted CoQ10 (µmol/mmol Chol)		0.18 ± 0.06 *	0.19 ± 0.06	0.17 ± 0.04	0.19 ± 0.07	F_(2, 79)_ = 0.14, *p* = 0.866
Diet Type	mixed	93%	91%	91%	95%	χ^2^_(2)_ = 0.61, *p* = 0.735
	vegetarian/vegan	7%	9%	9%	5%	
PA Levels	low	18%	11%	13%	29%	χ^2^_(4)_ = 5.43, *p* = 0.246
	moderate	21%	20%	22%	21%	
	high	61%	69%	65%	50%	
Sedentary Time (h/week)		42.5 ± 23.3	43.9 ± 22.5	38.8 ± 24.6	45.3 ± 22.5	F_(2, 120)_ = 1.52, *p* = 0.222
Work MET		4889 ± 9949	5114 ± 7306	4980 ± 10481	4602 ± 11382	F_(2, 120)_ = 1.83, *p* = 0.165
Transport MET		313 ± 1866	62 ± 234	703 ± 3005	97 ± 356	F_(2, 120)_ = 1.03, *p* = 0.361
Recreation MET		3395 ± 4448	3514 ± 3051	3235 ± 5113	3472 ± 4733	F_(2, 120)_ = 0.90, *p* = 0.409
Total MET		8598 ± 13526	8690 ± 7866	8918 ± 17276	8171 ± 12853	F_(2, 120)_ = 2.25, *p* = 0.110

* Total number of participants: 82. BMI: body mass index; PA: physical activity; MET: metabolic equivalent of task; CoQ10: Coenzyme Q10. Data are presented as percentage, median (with range), or mean ± standard deviation. Statistical tests: chi-square (χ^2^) and Kruskal–Wallis (F-values).

**Table 2:** Comparison of blood micronutrient levels among female participants separated into three age categories.

	Reference Range for Female Adults *	Females (*n* = 62)				Statistics and *p*-Values
		Total	18–34 Year	35–49 Year	50–64 Year	
Potassium (mg/L)	1484–1794	1687 ± 234	1724 ± 134	1616 ± 366	1718 ± 119	F_(2, 59)_ = 1.15, *p* = 0.323
Calcium (mg/L)	53.8–62.7	56.3 ± 4.1	55.8 ± 4.7	56.7 ± 3.2	56.4 ± 4.3	F_(2, 59)_ = 1.22, *p* = 0.301
Magnesium (mg/L)	29.8–37.5	32.8 ± 3.0	31.9 ± 3.1	32.1 ± 3.5	34.2 ± 1.9	F_(2, 59)_ = 5.24, *p* = 0.008
Copper (mg/L)	0.76–1.12	0.93 ± 0.20	1.01 ± 0.29	0.88 ± 0.12	0.92 ± 0.12	F_(2, 59)_ = 0.88, *p* = 0.419
Iron (mg/L)	423–520	482 ± 43	477 ± 48	479 ± 49	489 ± 34	F_(2, 59)_ = 0.75, *p* = 0.476
Zinc (mg/L)	4.88–6.67	5.80 ± 0.69	5.98 ± 0.63	5.47 ± 0.83	5.93 ± 0.51	F_(2, 59)_ = 1.99, *p* = 0.146
Selenium (µg/L)	101–170	138 ± 42	130 ± 29	135 ± 36	149 ± 54	F_(2, 59)_ = 2.06, *p* = 0.137
Manganese (µg/L)	5.91–12.7	10.33 ± 3.70	10.32 ± 4.20	11.17 ± 3.67	9.57 ± 3.20	F_(2, 59)_ = 1.38, *p* = 0.259
Molybdenum (µg/L)	0.5–1.6	1.12 ± 0.83	0.85 ± 0.37	1.27 ± 0.83	1.22 ± 1.06	F_(2, 59)_ = 1.67, *p* = 0.197
Vitamin B_6_ (µg/L)	16.4–80.4	38.2 ± 30.8	33.3 ± 13.3	29.6 ± 8.7	50.6 ± 47.6	F_(2, 59)_ = 1.73, *p* = 0.187
Vitamin B_9_ (ng/mL)	>5.38	8.88 ± 5.70	7.37 ± 4.61	8.04 ± 5.28	10.91 ± 6.50	F_(2, 57)_ = 1.67, *p* = 0.197
Vitamin B_12_ (pg/mL)	211–911	579 ± 877	451 ± 143	831 ± 1554	478 ± 158	F_(2, 58)_ = 0.24, *p* = 0.785
Vitamin D (nmol/L)	100–150	89.9 ± 37.2	84.1 ± 24.4	96.7 ± 42.2	89.1 ± 42.3	F_(2, 59)_ = 0.32, *p* = 0.728

* Values are based on reference norms for German-speaking populations [32]. Data are presented as mean (±standard deviation). Statistical test: Kruskal–Wallis (F-values).

**Table 3:** Comparison of blood micronutrient levels among male participants separated into three age categories.

	Reference Range for Male Adults *	Males (*n* = 61)				Statistics and *p*-Values
		Total	18–34 Year	35–49 Year	50–64 Year	
Potassium (mg/L)	1568–1908	1811 ± 113	1800 ± 86	1839 ± 108	1782 ± 131	F_(2, 58)_ = 1.10, *p* = 0.340
Calcium (mg/L)	50.3–59.8	53.5 ± 3.4	55.0 ± 2.4	53.1 ± 4.3	52.8 ± 2.4	F_(2, 58)_ = 3.02, *p* = 0.057
Magnesium (mg/L)	31.2–39.1	34.9 ± 2.8	34.3 ± 2.3	35.1 ± 2.8	35.0 ± 3.1	F_(2, 58)_ = 0.26, *p* = 0.773
Copper (mg/L)	0.7–0.94	0.79 ± 0.09	0.75 ± 0.09	0.79 ± 0.08	0.82 ± 0.09	F_(2, 58)_ = 3.36, *p* = 0.042
Iron (mg/L)	465–577	543 ± 34	546 ± 22	542 ± 40	541 ± 33	F_(2, 58)_ = 0.13, *p* = 0.874
Zinc (mg/L)	5.36–7.29	6.43 ± 0.68	6.12 ± 0.68	6.42 ± 0.62	6.69 ± 0.69	F_(2, 58)_ = 2.23, *p* = 0.117
Selenium (µg/L)	101–168	136 ± 35	144 ± 61	131 ± 25	137 ± 18	F_(2, 58)_ = 0.93, *p* = 0.402
Manganese (µg/L)	5.39–11.2	8.29 ± 2.13	8.37 ± 1.64	8.38 ± 2.74	8.13 ± 1.56	F_(2, 58)_ = 0.04, *p* = 0.958
Molybdenum (µg/L)	0.45–1.56	1.60 ± 4.23	1.33 ± 0.66	2.23 ± 6.45	0.98 ± 0.67	F_(2, 58)_ = 2.24, *p* = 0.115
Vitamin B_6_ (µg/L)	16.4–80.4	36.5 ± 22.3	37.2 ± 16.7	41.4 ± 28.6	29.7 ± 14.5	F_(2, 58)_ = 1.88, *p* = 0.161
Vitamin B_9_ (ng/mL)	>5.38	10.02 ± 4.92	10.95 ± 5.30	9.47 ± 4.53	10.04 ± 5.26	F_(2, 58)_ = 0.29, *p* = 0.746
Vitamin B_12_ (pg/mL)	211–911	491 ± 247	567 ± 402	458 ± 120	478 ± 222	F_(2, 58)_ = 0.27, *p* = 0.767
Vitamin D (nmol/L)	100–150	99.8 ± 49.9	106.1 ± 50.2	98.4 ± 57.1	97.0 ± 40.9	F_(2, 58)_ = 0.21, *p* = 0.810

* Values are based on reference norms for German-speaking populations [32]. Data are presented as mean (±standard deviation). Statistical test: Kruskal-Wallis (F-values).

## Data Availability

The original contributions presented in the study are included in the article/supplementary material, further inquiries can be directed to the corresponding author/s.

## Supplementary Materials

The following supporting information can be downloaded at: https://www.mdpi.com/article/10.3390/clinpract14020034/s1.
