# Supplementary material for: Health Patterns across Adulthood: An Age-Based Investigation of the Nutritional Status, Homocysteine, and CoQ10 of Bank Staff
Source: Clin Pract. 2024 Mar 14;14(2):443–60. doi: 10.3390/clinpract14020034 (PMC10961793; doi:10.3390/clinpract14020034)
Supplement: Supplementary file 1 [file clinpract-14-00034-s001.zip › clinpract-2852103-supplementary.pdf]

Dear participant,

Thank you for taking part in our study! Your precise and accurate answers are crucial for the success of our study. Please answer the following questions and provide us with detailed information.

Name: ________________________

Code: ______

Contact information (Phone and address):

____

Age: ______ years

Gender: [ ] male [ ] female [ ] other

Weight: ______ kg

Height: ______ cm

Name and address of bank: ____

Working position in bank: _____________________

Diet type: Please select the option that best describes your current diet type:

[ ] Omnivore: Eats both animal and plant foods.

[ ] Vegetarian: Does not eat meat (including poultry and fish), but consumes dairy products and/or eggs.

[ ] Vegan: Does not eat animal products, including meat, dairy, eggs and honey.

If you have chosen "vegetarian" or "vegan", please briefly explain how long you have been following this diet and what the main reasons for this diet are:

____

Medical Histroy:

Please check one of the following diseases that you have been diagnosed with or are currently suffering from:

[ ] High blood pressure

[ ] Diabetes

[ ] Cardiovascular disease

[ ] Cancer

[ ] Arthritis

[ ] Gastrointestinal disease

[ ] Mental illness

If you have ticked one of the above diseases, please indicate the disease(s) and provide any additional information you consider relevant:

____

Have you ever had surgery? If yes, please state the type and date:

____

Are you currently taking any prescription medication? If yes, please list:

____

Are there any other significant medical conditions or health events in your medical history that you think we should know about? Please list:

____

Global Physical Activity Questionnaire GPAQ (by WHO)

Physical Activity

Next I am going to ask you about the time you spend doing different types of physical activity in a typical week. Please answer these questions even if you do not consider yourself to be a physically active person.

Think first about the time you spend doing work. Think of work as the things that you have to do such as paid or unpaid work, study/training, household chores, harvesting food/crops, fishing or hunting for food, seeking employment. *[Insert other examples if needed].* In answering the following questions 'vigorous-intensity activities' are activities that require hard physical effort and cause large increases in breathing or heart rate, 'moderate-intensity activities' are activities that require moderate physical effort and cause small increases in breathing or heart rate.

Questions		Response	Code
**Activity at work**			
1	Does your work involve vigorous-intensity activity that causes large increases in breathing or heart rate like *[carrying or lifting heavy loads, digging or construction work*] for at least 10 minutes continuously?<br>*[INSERT EXAMPLES] (USE SHOWCARD)*	Yes 1<br>No 2 *If No, go to P 4*	P1
2	In a typical week, on how many days do you do vigorous-intensity activities as part of your work?	Number of days └─┘	P2
3	How much time do you spend doing vigorous-intensity activities at work on a typical day?	Hours : minutes └─┴─┘ : └─┴─┘<br>hrs mins	P3<br>(a-b)
4	Does your work involve moderate-intensity activity that causes small increases in breathing or heart rate such as brisk walking *[or carrying light loads*] for at least 10 minutes continuously?<br>*[INSERT EXAMPLES] (USE SHOWCARD)*	Yes 1<br>No 2 *If No, go to P 7*	P4
5	In a typical week, on how many days do you do moderate-intensity activities as part of your work?	Number of days └─┘	P5
6	How much time do you spend doing moderate-intensity activities at work on a typical day?	Hours : minutes └─┴─┘ : └─┴─┘<br>hrs mins	P6<br>(a-b)
**Travel to and from places**			
The next questions exclude the physical activities at work that you have already mentioned.<br>Now I would like to ask you about the usual way you travel to and from places. For example to work, for shopping, to market, to place of worship. [insert other examples if needed]			
7	Do you walk or use a bicycle (*pedal cycle*) for at least 10 minutes continuously to get to and from places?	Yes 1<br>No 2 *If No, go to P 10*	P7
8	In a typical week, on how many days do you walk or bicycle for at least 10 minutes continuously to get to and from places?	Number of days └─┘	P8
9	How much time do you spend walking or bicycling for travel on a typical day?	Hours : minutes └─┴─┘ : └─┴─┘<br>hrs mins	P9<br>(a-b)
**Recreational activities**			
The next questions exclude the work and transport activities that you have already mentioned.<br>Now I would like to ask you about sports, fitness and recreational activities (leisure), [insert relevant terms].			
10	Do you do any vigorous-intensity sports, fitness or recreational (*leisure*) activities that cause large increases in breathing or heart rate like [*running or football,]* for at least 10 minutes continuously?<br>*[INSERT EXAMPLES] (USE SHOWCARD)*	Yes 1<br>No 2 *If No, go to P 13*	P10
11	In a typical week, on how many days do you do vigorous-intensity sports, fitness or recreational (*leisure*) activities?	Number of days └─┘	P11
12	How much time do you spend doing vigorous-intensity sports, fitness or recreational activities on a typical day?	Hours : minutes └─┴─┘ : └─┴─┘<br>hrs mins	P12<br>(a-b)

Continued on next page

Global Physical Activity Questionnaire GPAQ (by WHO),
Continued

**Physical Activity (recreational activities)** contd.			
**Questions**		**Response**	**Code**
13	Do you do any moderate-intensity sports, fitness or recreational *(leisure)* activities that causes a small increase in breathing or heart rate such as brisk walking,(*cycling, swimming, volleyball*)for at least 10 minutes continuously?<br>*[INSERT EXAMPLES] (USE SHOWCARD)*	Yes 1<br>No 2 *If No, go to P16*	P13
14	In a typical week, on how many days do you do moderate-intensity sports, fitness or recreational (*leisure*) activities?	Number of days └─┘	P14
15	How much time do you spend doing moderate-intensity sports, fitness or recreational (*leisure*) activities on a typical day?	Hours : minutes └─┴─┘ : └─┴─┘<br>hrs mins	P15 (a-b)
**Sedentary behaviour**			
The following question is about sitting or reclining at work, at home, getting to and from places, or with friends including time spent [sitting at a desk, sitting with friends, travelling in car, bus, train, reading, playing cards or watching television], but do not include time spent sleeping.<br>*[INSERT EXAMPLES] (USE SHOWCARD)*			
16	How much time do you usually spend sitting or reclining on a typical day?	Hours : minutes └─┴─┘ : └─┴─┘<br>hrs min s	P16 (a-b)
